# Role of PI3K in the Progression and Regression of Atherosclerosis

**DOI:** 10.3389/fphar.2021.632378

**Published:** 2021-03-09

**Authors:** Yunyun Zhao, Yongjiang Qian, Zhen Sun, Xinyi Shen, Yaoyao Cai, Lihua Li, Zhongqun Wang

**Affiliations:** ^1^ Department of Pathology, Affiliated Hospital of Jiangsu University, Zhenjiang, China; ^2^ Department of Cardiology, Affiliated Hospital of Jiangsu University, Zhenjiang, China

**Keywords:** PI3K, signaling pathways, drug therapy, atherosclerosis, plaque

## Abstract

Phosphatidylinositol 3 kinase (PI3K) is a key molecule in the initiation of signal transduction pathways after the binding of extracellular signals to cell surface receptors. An intracellular kinase, PI3K activates multiple intracellular signaling pathways that affect cell growth, proliferation, migration, secretion, differentiation, transcription and translation. Dysregulation of PI3K activity, and as aberrant PI3K signaling, lead to a broad range of human diseases, such as cancer, immune disorders, diabetes, and cardiovascular diseases. A growing number of studies have shown that PI3K and its signaling pathways play key roles in the pathophysiological process of atherosclerosis. Furthermore, drugs targeting PI3K and its related signaling pathways are promising treatments for atherosclerosis. Therefore, we have reviewed how PI3K, an important regulatory factor, mediates the development of atherosclerosis and how targeting PI3K can be used to prevent and treat atherosclerosis.

## Introduction

Atherosclerosis is a leading cause of vascular death worldwide (Herrington et al., 2016). The pathophysiological processes of atherosclerosis include endothelial dysfunction, lipid deposition, foam cell formation, plaque formation, increased plaque vulnerability and plaque rupture. Atherosclerosis is a complex pathological process caused by activation of the arterial endothelium by various risk factors. Many studies have shown that endothelial cell injury initiates atherosclerosis. After injury, endothelial cells secrete chemokines and inflammatory molecules such as monocyte chemoattractant protein-1 (MCP-1). Endothelial cells and smooth muscle cells (SMC) produce macrophage colony-stimulating factor, which stimulates the differentiation of monocytes into macrophages. These chemokines attract immune cells, especially T lymphocytes and monocytes (Gisterå and Hansson, 2017). Low-density lipoprotein (LDL) in the blood accumulates at endothelial cell injury sites and penetrates into the endothelium through fissures, where it is modified to oxidized low-density lipoprotein (ox-LDL), acetylated low-density lipoprotein, and enzyme-modified low-density lipoprotein. All of these LDLs can enter mononuclear macrophages and smooth muscle cells in a positive feedback loop, thus inducing the formation of foam cells. Ox-LDL can in turn block the migration of mononuclear macrophages and foam cells out of the injury site, thus accelerating the accumulation of foam cells and the formation of lipid plaques (Di Pietro et al., 2016).

Ox-LDL autotoxicity and cytokine induction in plaques exacerbate the inflammatory response, leading to endothelial cell apoptosis, and platelets gradually aggregate and adhere to the damaged endothelium. The release of growth factors by phagocytes, endothelial cells, and platelets attached to the injury site of endothelial cells stimulates the migration and proliferation of SMCs from the tunica media to the intima These SMCs synthesize extracellular matrix (ECM) to form complex fibrotic plaques. Proliferation of SMCs leads to the thickening of the intima and stenosis of the lumen. Foam cell fragments and necrotic substances cause macrophages to release a large amount of proinflammatory cytokines and reactive oxygen species (ROS).

In addition, protein hydrolases produced by macrophages decompose the ECM and promote plaque instability. Phenotypic changes in cytokine-stimulated endothelial cells in plaques lead to trophoblast-mediated angiogenesis, further exacerbating endothelial cell penetration, cholesterol lipid accumulation and intraplaque hemorrhage; increasing plaque vulnerability; and promoting plaque development and acute coronary events. Foam cell apoptosis, cholesterol crystallization and other events contribute to plaque lipid core formation and are key factors in plaque rupture. Autophagy in smooth muscle leads to the thinning of the fibrous cap, and the transition of SMCs from a contractile to a synthetic phenotype leads to smooth muscle calcification and the formation of calcification foci, which exacerbate the development of atherosclerosis.

Phosphatidylinositol 3 kinase (PI3K) is an intracellular kinase located on the medial side of the cell that regulates the survival, proliferation, migration, differentiation, transcription and translation of cells in the context of atherosclerosis through the activation of signaling pathways; PI3K also plays an important role in the progression and regression of atherosclerosis. Endothelial cell apoptosis, lipid accumulation and transport, macrophage autophagy, phenotypic transition, excessive smooth muscle proliferation, and the expression of adhesion molecules involved in the inflammatory response all involve PI3K signaling. Hence, PI3K and its signaling pathways are likely to be ideal targets for the treatment of atherosclerosis. From basic and clinical perspectives, this review focuses on PI3K in the progression, regression and treatment of atherosclerosis.

## PI3K and Its Signaling Pathways

### PI3K

PI3K, a key molecule in the signal transduction pathways initiated by the binding of extracellular signals to cell surface receptors, has serine/threonine kinase activity and phosphatidylinositol kinase activity. The PI3K family includes eight catalytic subtypes, classified into three categories according to sequence homology and *in vitro* substrates. Among them, the most widely studied has been the class I PI3Ks, which can be further divided into the class IA and IB. Class IA molecules are heterodimers composed of p110 catalytic subunits and p85 regulatory subunits. The three subtypes of p110 catalytic subunits (α, ß and δ), are encoded by the PIK3CA, PIK3CB and PIK3CD genes, respectively. Class IB PI3Ks consist of the catalytic subunit p110γ; the regulatory subunits p110α and p110β are universally expressed, while p110λ and p110δ are enriched in immune cells.

Class IA PI3Ks are activated by multiple cell surface receptors. The phosphorylation of phosphatidylinositol 4,5-bisphosphate [PI(4,5)P2] forms phosphatidylinositol 3,4,5-trisphosphate [PI(3,4,5)P3] via growth factor receptors and G protein-coupled receptors. This phospholipid acts as a second messenger for the recruitment of cytoplasmic proteins to a specific plasma membrane or intimal position. Regulatory subunits contain SH2 and SH3 domains, and target proteins contain corresponding binding sites. In normal cells, PI(3,4,5)P3 is briefly induced by growth factor stimulation and is rapidly metabolized by lipid phosphatases, including phosphatase and tensin homolog (PTEN), terminating PI3K signaling by removing the 3′ phosphoric acid from PI(3,4,5)P3. In addition, the phosphatase SH2-containing inositol phosphatase removes the 5′ phosphoric acid from PI(3,4,5)P3, converting PI(3,4,5)P3 to PI(3,4)P2 and thereby blocking the activation of its downstream effector molecules (Durrant and Hers, 2020).

The physiological function of class II PI3Ks has not been fully elucidated; the three members of this class, PI3KC2α, PI3KC2β and PI3KC2γ, are involved in the production of PI (3,4) P2 through the use of PI (4)P as a catalytic substrate. Class III PI3Ks consists of a regulatory subunit (Vps15; also known as p150) and a catalytic subunit (Vps34). Class III PI3Ks, which are homologous to the yeast protein Vps34, are evolutionarily conserved and can only use only PtdIns as a substrate to produce PtdInsP3 during catalysis. Moreover, the induction of autophagy requires Vps34, Vps15, and Beclin as components of the Vps34 complex. Similar to class I PI3Ks, Vps34 can control cell growth by regulating the mammalian rapamycin complex 1 (mTORC1)/ribosomal protein S6 kinase 1 (S6K1) pathway, which regulates protein synthesis in response to amino acid availability.

PI3K activation largely involves substrates close to the medial side of the plasma membrane. Multiple growth factors and signaling complexes, including fibroblast growth factor, vascular endothelial growth factor (VEGF), hepatocyte growth factor, angiotensin I and insulin, initiate PI3K activation.

### PI3K and Its Downstream Effectors

AKT, known as protein kinase B (PKB), is the main effector that is downstream of PI3K. PI3K activation forms PIP3 on the cell membrane. PIP3 is a second messenger that activates downstream proteins, among the most important of which is phosphoinositide-dependent protein kinase-1 (PDK1), which controls the activation of PKB/AKT signal transduction. PIP3 binds the intracellular signaling proteins Akt and PDK1 and the promotes phosphorylation of Akt at Thr308. However, Akt activation, also requires its phosphorylation at Ser473 by mTORC2. Activated Akt activates or inhibits the downstream target proteins Bad, Caspase9, nuclear factor-kappa B (NF-κ B), and glycogen synthase kinase-3β (GSK3β) through phosphorylation, thus regulating cell proliferation, differentiation, apoptosis and migration. Akt affects the cell cycle and glucose metabolism through GSK3β, regulating cell growth and survival via mTORC1, S6K1and 4-E-binding proteins to control the mechanisms of translation. In addition, Akt regulates cell survival by phosphorylating forkhead the human rhabdomyosarcoma transcription factor to inhibit the translation of preapoptotic genes, such as cell death Bcl-2 antagonist (BAD), Bcl-2-interacting cell death mediator (BIM), and Fas ligands (FasL).

In addition to Akt, effectors downstream of PI3K include Ras-related C3 botulinum toxin substrate 1 (Rac1) and Protein kinase Cζ (PKCζ), but of the many of PI3K signaling pathways, the PI3K/Akt pathway is most closely related to atherosclerosis. This paper also focuses on the PI3K/Akt pathway.

## PI3K and Atherosclerotic Plaques

### Effects of PI3K on Atherosclerotic Plaque Formation

Atherosclerotic plaque formation is a typical feature of atherosclerosis. Activation of PI3K/Akt signaling can induce monocyte chemotaxis, macrophage migration, increased intracellular lipid accumulation, neovascularization, SMC proliferation and dysfunction in lesions, all of which are involved in plaque formation.

Fetuin-A exerts stimulatory effects on vascular SMC (VSMC) proliferation and ECM expression via the PI3K/AKT/c-Src/NF-kB/ERK1/2 pathways, which can accelerate the development of atherosclerosis (Naito et al., 2016). Angiopoietin 1 induces monocyte chemotaxis, and PI3K is an indispensable part of this process. Research has shown that macrophages lacking PI3K cannot migrate in response to chemokine stimulation. Ox-LDL is an independent risk factor for atherosclerosis that induces growth factors and cytokines, triggers PI3K signaling in macrophages/foam cells, and promotes foam cell formation. Platelet activating factor receptors and CD36 mediate atherosclerosis by activating the human monocyte/macrophage PI3K/Akt pathway to activate p38 and c-Jun-N-terminal kinase - mitogen-activated protein kinase (MAPK), increasing ox-LDL uptake and promoting foam cell formation (Rios et al., 2012). Ox-LDL promotes the proliferation of SMCs through PI3K/Akt/mTOR, thickening the intima of the arterial wall and enlarging the plaque (Brito et al., 2009).

**FIGURE 1:**
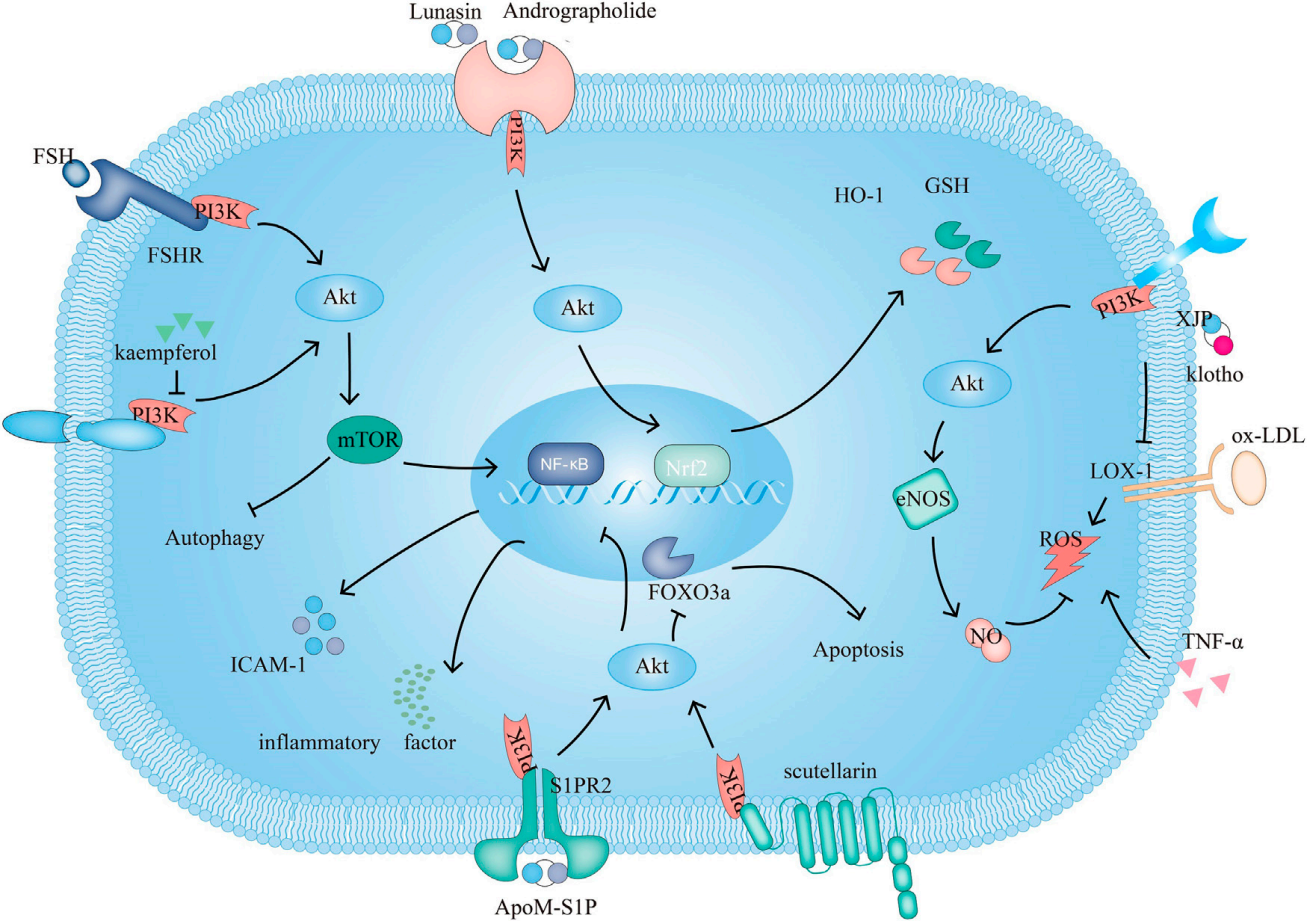
The PI3K signaling pathway and related target drugs in endothelial cells. The PI3K signaling pathway affects three main factors in endothelial cells: 1. Survival and apoptosis. ① Kaempferol enhances autophagy by inhibiting PI3K/Akt/mTOR and inhibiting apoptosis. ② Scutellarin exerts antiapoptotic effects through PI3K/Akt/FOXO3a. 2. Inflammation. ③ ApoM-S1P/S1PR2 activates PI3K/Akt, which suppresses ox-LDL-induced inflammation. ④FSH/FSHR activate PI3K/Akt/mTOR/NF-κB, increase ICAM-1 expression and promote monocyte-endothelial cell adhesion 3. Oxidation. ⑤ Klotho, XJP can downregulate the expression of LOX-1 and by PI3K/Akt/eNOS/NO-mediated inhibition of the increase in ROS, resisting apoptosis. ⑥Andrographolide increases GSH and HO-1 and inhibits TNF-α-induced ROS production through PI3K/Akt/Nrf2 and PI3K/Akt/AP-1-mediated upregulation of expression. Lunasin increases HO-1 through PI3K/Akt/Nrf2.

In vascular inflammation, NF-κB signal transduction is also an important regulator of endothelial cell adhesion molecules. Monocyte-endothelial cell adhesion is thought to be the primary promoter of inflammatory vascular diseases such as atherosclerosis. Follicle stimulating hormone promotes the expression of VCAM-1 (Figure 1) through the PI3K/Akt/mTORC2/NF-κB pathway, thereby promoting monocyte adherence to human endothelial cells, which could contribute to the follicle-stimulating hormone-induced exacerbation of atherosclerosis development in postmenopausal women (Li et al., 2017). Leukotriene B4 is partially up-regulated by Akt/NF-κB expression of the pro-inflammatory cytokines involved in atherosclerosis (Sánchez-Galán et al., 2009). JAM-1, a cell adhesion molecule that is selectively localized at the tight junctions of endothelial cells, plays an important role in the adhesion of platelets to inflammatory endothelial cells. Apolipoprotein C3, promotes JAM-1 expression through the PI3K/IKK2/P65 signaling pathway and promotes inflammation during atherosclerosis (Dai et al., 2019). Platelet-activating factor increases the enzymatic activity of phosphatase nonreceptor type 2 and mediates IL-6 mRNA expression through the PI3K/Akt pathways, which are involved in the inflammatory response (Hamel-Cote et al., 2019).

**FIGURE 2:**
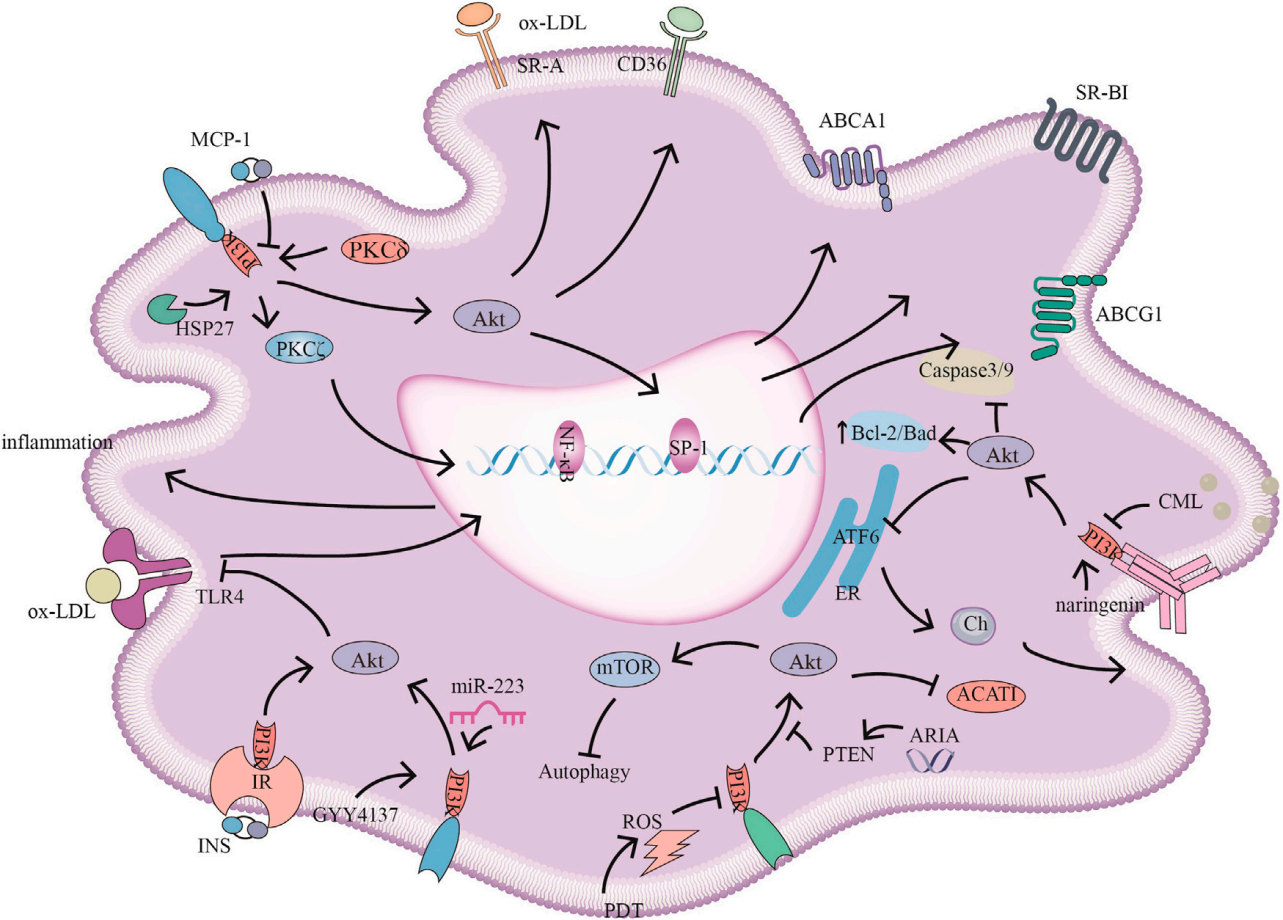
PI3K signaling pathways and related target drugs in macrophages. PI3K signaling pathways have the following main effects in macrophages: 1. Cholesterol ester formation and cholesterol efflux. ①MCP-1 inhibits ABCA1, ABCG1, and SR-BI expression by inhibiting PI3K/Akt activity. ②HSP27 act through PI3K/PKCζ/SP1 to promote ABCA1 expression. ③ Reductions in ARIA decrease PTEN activity, promote PI3K/Akt, and inhibit ACAT1 activity and cholesterol ester accumulation. ④ Naringenin inhibits endoplasmic reticulum-associated ATF6 activity by activating PI3K/Akt and promoting cholesterol efflux in macrophages. ⑤ PKCδ activates PI3K/Akt, increases SR-A and CD36 expression and promotes foam cell formation 2. The inflammatory response ⑥ INS/IR, GYY4137, and miR-223 attenuate the inflammatory response by activating PI3K/Akt, and negatively regulate TLR4/NF-κB; GYY4137, and miR-223, also inhibiting TLR4-mediated lipid deposition. 3. Autophagy and apoptosis. ⑦ CML promotes apoptosis by inhibiting PI3K/Akt/Caspase3/9 and PI3K/Akt/Bad. ⑧ PDT-induced ROS inhibits PI3K/Akt/mTOR, promoting autophagy and reducing the inflammatory response.

PI3K plays a role in mediating macrophage foam cell formation. Leptin accelerates cholesterol ester accumulation in human monocyte-derived macrophages and inhibits cholesterol efflux via PI3K, increasing ACAT-1 expression (Hongo et al., 2009). MCP-1 (Figure 2)reduces the expression of ATP-binding cassette transporter A1 (ABCA1), ABCG1 and Scavenger Receptor class B type I (SR-BI), resulting in increased lipid accumulation and foam cell formation (Huang et al., 2013; Cavelier et al., 2006). Similarly, pregnancy-associated plasma protein A (PAPP-A) downregulates liver X receptor expression through the IGF-1/PI3K/Akt signaling pathway, in turn reducing ABCA1, ABCG1, and SR-B1 expression and cholesterol efflux in macrophage-derived foam cells and promoting plaque formation (Tang et al., 2012). PI3K is regulated by its upstream signals; on the one hand, it can promote intracellular lipid deposition and lead to the formation of foam cells and atherosclerotic plaques, and on the other hand, it can reduce the expression of lipid transporters and reduce the efflux of intracellular cholesterol.

### Effects of PI3K on the Stability of Atherosclerotic Plaques

Atherosclerotic plaque stability is closely related to atherosclerotic progression. Plaque rupture is associated with many complications; ruptured plaques obstruct blood vessels, leading to thrombosis. The inflammatory response is one of the most important intrinsic factors associated with plaque vulnerability. Studies have shown that p110γ deletion reduces the infiltration of monocytes and T cells, and a reduction in the inflammatory response increases plaque stability (Fougerat et al., 2008). Macrophages in plaques constitute a key factor in plaque-associated inflammation. During plaque rupture, macrophages secrete many inflammatory molecules. In addition, macrophages secrete matrix metalloproteinases to degrade the ECM, causing the fiber cap to become thin and enhancing plaque vulnerability (Sakakura et al., 2013). Recent studies have shown that promoting autophagy in macrophages, reducing macrophage infiltration in plaques, and promoting the stability of vulnerable plaques haves become new therapeutic strategies for atherosclerosis.

Autophagy, a mode of cell death, plays an important role in regulating the development of atherosclerosis and stability of atherosclerotic plaques. Autophagy is the process by which damaged organelles and macromolecules are degraded by lysosomes under the regulation of autophagy-related genes. Typical, moderate autophagy is an important protective mechanism by which atherosclerotic plaque cells protect against antioxidant stress and inflammation and an important determinant of plaque stability. Inadequate or excessive autophagy affects the development of plaques. In most atherosclerosis cases, autophagy can inhibit apoptosis (Mialet-Perez and Vindis, 2017).

PI3K/AKT/mTOR signaling pathway-mediated autophagy has been confirmed in several studies. mTOR is an atypical serine/threonine protein kinase and an important effector molecule downstream of the PI3K/AKT signaling pathway. mTOR, which plays a key role in cell growth, proliferation, metabolism and the improvement of cardiac function, consist of two subtypes: mTORC1 and mTORC2. mTORC1 is involved in regulating cell growth, proliferation, apoptosis, autophagy, energy metabolism, and S6 kinase 1 by phosphorylating the ribosomal protein S6K1, and it promotes mRNA biosynthesis, ribosomal protein translation, cell growth and metabolism. mTORC2 is involved in cytoskeletal protein construction, survival and cell migration.

Reducing miR-155 (Zhai et al., 2014) andmiR-135b can effectively inhibit the PI3K/Akt/mTOR signaling pathways, enhance macrophage autophagy, and reduce plaque infiltration by macrophages, which in the early stage of atherosclerosis reduces the accumulation of foam cells and inhibits the formation and development of plaques (Wu et al., 2020). The latest findings indicate that the use of nanoparticles encapsulating chlorin e6-mediated photodynamic therapy (PDT) activates autophagy (Figure 2) through the generation of ROS and inhibiting the PI3K/AKT/mTOR signaling pathways, and expression of proinflammatory factors in peritoneal M1 macrophages, thus reducing inflammation and increasing plaque stability (Han et al., 2019).

In addition to the induction of macrophage autophagy, a reduction in intracellular lipid deposition and the direct inhibition of inflammatory factors can reduce plaque vulnerability. Toll-like receptor 4 (TLR4) was found to be necessary for the uptake of ox-LDL by mouse macrophages and their differentiation into foam cells. Activation of TLR4 increased foam cell formation, lipid deposition, and the level of inflammatory cytokines, ultimately affecting plaque rupture. PI3K/Akt signaling was recently suggested to negatively regulate TLR4-mediated inflammation. Zheng Y et al. first confirmed that morpholin-4-ium-methoxyphenyl-morpholino-phosphinodithioate (GYY4137) improves foam cell formation *in vitro* through the PI3K/AKT and TLR4 pathways and reduces the expression of proinflammatory cytokines, thus reducing the source of foam cells and lipid volume in unstable plaque tissues (Zheng et al., 2020). The NF-κB signaling pathway has been shown in several studies to be involved in the inflammation of atherosclerotic plaques. It also acts as a downstream effector of TLR4. Wang et al. demonstrated that tanshinone IIA and astragaloside IV stabilized vulnerable plaques in ApoE (−/−) mice fed a high-fat diet, through the PI3K/Akt/TLR4/NF-κB signaling pathway, which had an anti-inflammatory effect and inhibited the expression of MMP-9. Compared with ApoE (−/−) model mice, model mice treated with the a combination of tanshinone IIA and astragaloside IV also showed a significant reduction in the lipid area of the right common carotid artery; an increased collagen content, thickening the fibrous cap; and reduced ox-LDL-induced accumulation of cytoplasmic lipid droplets in RAW 264.7 macrophages (Wang et al., 2020). Similarly, quercetin attenuates the inflammatory response by inhibiting the activation of TLR4/MyD88/NF-κB signaling through the PI3K/Akt pathway, promoting the stability of atherosclerotic plaques (Endale et al., 2013).

The latest research by our group showed that the inhibition of PI3K/Akt can enhance the plaque vulnerability by promoting the apoptosis of foam cells. In advanced atherosclerosis, foam cell apoptosis increases the plaque necrotic core, the inflammatory response and vascular vulnerability due to the weakening of endocytic clearance mechanisms. We used sufficient experimental evidence to demonstrate that the advanced glycation end-product Nε-carboxymethyl-lysine (CML) promotes foam cell apoptosis (Figure 2) in diabetic atherosclerotic plaques through classic apoptosis pathways (Bcl-2/Bax, caspase-3 and caspase-9) in a concentration-dependent manner, and we suggested that the underlying mechanism involved inhibition of the PI3K/Akt signaling pathways (Wang et al., 2019). Macrophage SR-BI binds phosphatidylserine on apoptotic cells and mediates the endocytosis of apoptotic cells in atherosclerotic lesions through the Src/PI3K/Rac1 pathway, reducing plaque necrosis and inflammation. Hematopoietic SR-BI deficiency leads to atherosclerotic necrosis, decreased collagen content and fibrous cap thickness (Tao et al., 2015).

Endothelial cell injury and apoptosis lead to an inflammatory response and platelet aggregation, resulting in a decrease in plaque stability and promoting atherosclerotic lesions and thrombosis. Activating the PI3K/Akt/endothelial nitric oxide synthase (eNOS) pathway induces endothelial cells to release nitric oxide (NO), which is key to maintaining vascular homeostasis as it induces vasodilation and exhibits vascular protection (Malekmohammad et al., 2020). VSMC apoptosis leads to thinning of the protective fibrous cap and enhanced plaque vulnerability in early and late lesions. Soluble N-cadherin, a kind of cell adhesion molecule, attenuates VSMC apoptosis and inhibits plaque instability through PI3K/Akt (Lyon et al., 2009). IFN-γ in atherosclerotic plaques promotes VSMC apoptosis, in part because IFN-γ promotes Fas migration to the cell surface, a process that involves PI3K/Akt and JAK-2/STAT1 (Rosner et al., 2006).

In recent years, intraplaque angiogenesis has been considered to be an important risk factor in the transformation of stable plaques into unstable plaques. The normal vascular network exists in only the outer 1/3 of the outer and middle membranes, and in atherosclerotic regions, the blood vessels are more abundant and extend to the intima. On the one hand, neovascularization in the plaque involves a simple endothelial cell tube, lacking tight connections, with an incomplete basement membrane, unsupported by surrounding connective tissue; imperfect development of the vascular wall; and leakage of a large number of blood cells into the plaque, resulting in hematoma, increased lumen stenosis or even lumen occlusion and increased vulnerability. On the other hand, neovascularization provides a pathway for inflammatory cells and red blood cells to enter the plaque, promoting the maintenance of a chronic inflammatory state in the plaque intima (Sakakura et al., 2013).

VEGF is an angiogenic growth factor closely related to neovascularization, macrophage aggregation and instability within atherosclerotic plaques. In endothelial cells, PI3K activates eNOS and thus drives VEGFR-dependent angiogenesis. A recent study found that PI3K is a major regulator of developmental angiogenesis and VEGF-dependent endothelial cellproduction. PI3K is activated mainly by G-protein-coupled receptor stimulation and plays a crucial role in repairing angiogenesis. Zong et al. demonstrated that CD147 induced the upregulation of VEGF in U937-derived foam cells, further promoting intraplaque angiogenesis (Zong et al., 2016).

Immature plaque microvessels increase macrophage accumulation and the amount of oxygen and nutrients. These factors then lead to atherosclerotic plaque formation and facilitate the entry of leukocytes and red blood cells into atherosclerotic plaques. Therefore, monitoring and controlling angiogenesis are essential for the treatment of atherosclerosis. These studies have suggested that inhibiting the PI3K signaling pathways could reduce angiogenesis in plaques to some extent.

### Effects of PI3K on the Calcification of Atherosclerotic Plaques

Recent studies have shown that plaque calcification is also a regulated, active metabolic process similar to bone formation, rather than just a passive process consisting of calcium deposition and vascular degeneration, as had previously been proposed (Johnson et al., 2006). Many molecular mechanisms are involved in plaque calcification, such as the osteogenic differentiation of VSMCs, apoptosis, oxidative stress, inflammation and abnormal calcium and phosphorus metabolism (Durham et al., 2018). PI3K/Akt signaling plays an important role in regulating cell differentiation and is thus a classic signaling pathways associated with plaque calcification.

Phenotypic changes in (VSMCs) play an important role in the active regulation of plaque calcification. Osteogenic differentiation of SMCs is the most important cytopathological basis for arterial calcification. A study showed that PI3K/Akt is involved in the osteogenic differentiation of VSMCs. Sustainable expression of the calcification-related nuclear factors NF -κB receptor activator ligand (RANKL) and nuclear binding factor α1 (Cbfα, Runx2), osteopontin (OPN), osteocalcin, alkaline phosphatase and osteoprotegerin (OPG) in SMCs occurs following phenotypic transformation. These proteins regulate bone matrix formation and are involved in calcification (Iyemere et al., 2006).

**FIGURE 3:**
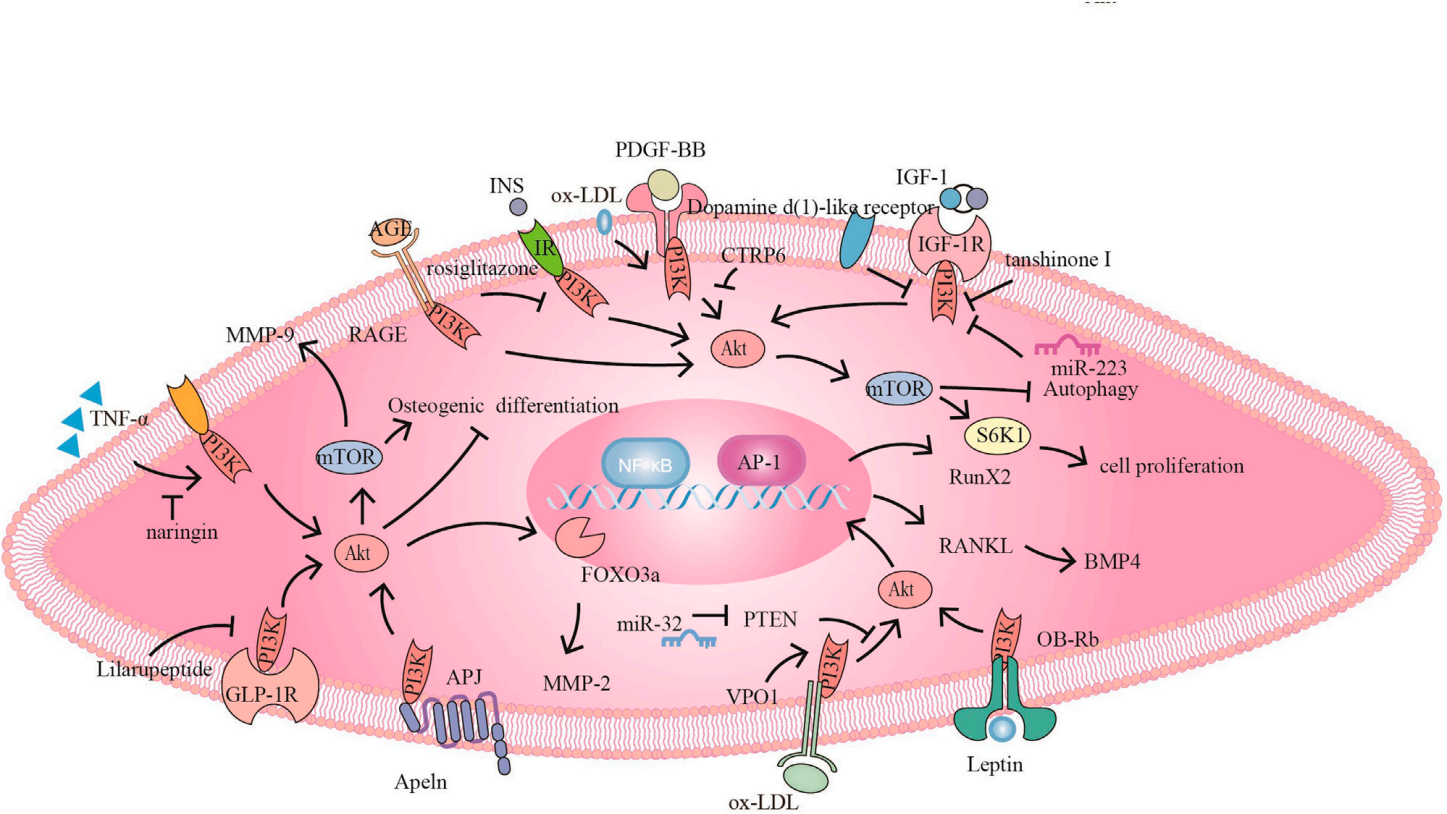
The PI3K signaling pathway and related target drugs in vascular smooth muscle cells. The PI3K signaling pathway has three main effects on vascular smooth muscle cells: 1. Proliferation and migration of smooth muscle cells. ① PDGF-BB activates PI3K/Akt/mTOR, promoting SMC proliferation and migration, while CTRP6 blocks this pathway ② Naringin inhibits TNF-α-induced PI3K/Akt/mTOR signaling, reduces MMP-9 secretion and inhibits cell proliferation and migration. ③ Tanshinone I and dopamine D1-like receptors inhibit proliferation by inhibiting IGF-1/IGF-1R signaling. ④ Apelin promotes migration through Apelin/APJ-mediated activation of PI3K/Akt/FOXO3a/MMP-2; furthermore, bone differentiation occurs through the inhibition of PI3K/Akt. ⑤ Ox-LDL promotes proliferation via PI3K/Akt/mTOR.⑥ Rosiglitazone inhibits INS/IR to activate PI3K/Akt/mTOR/P70S6K and promote proliferation. AGE/RAGE also promotes proliferation through PI3K/Akt 2. Osteogenic differentiation of smooth muscle cells⑦ VPO1 promotes ox-LDL-induced vascular calcification through the PI3K/AKT/Runx2 signaling pathway ⑧ miR-32 inhibits PTEN activity, promotes PI3K/Akt/RunX2 activation and exacerbates vascular calcification. ⑨ Lilaru peptide inhibits osteogenic differentiation by inhibiting PI3K/Akt/mTOR/S6K1 in combination with GLP-1R. ⑩Leptin promotes osteogenic differentiation via OB-Rb/PI3K/Akt/RANKL-BMP4. 3. Smooth muscle derived foam cells ⑪ miR-223 inhibits IGF-1/PI3K/Akt and Promote autophagy, reducing foam cell formation.

Runx2 is an osteoblast differentiation-associated transcription factor that plays an important role in mediating the osteogenic differentiation of VSMCs *in vitro* and *in vivo*. Vascular peroxidase 1 (VPO1) promotes ox-LDL-induced vascular calcification through the PI3K/AKT/Runx2 signaling pathway. VPO1 could be an endogenous regulator of vascular calcification, and targeting VPO1 (Figure 3) may be a novel strategy to prevent and treat vascular calcification (Tang et al., 2015); miR-32 (Figure 3) lowers PTEN l levels, in turn activating the PI3K/Akt signaling pathways to promote Runx2 expression in mouse VSMCs, thereby promoting cellular calcification (Liu et al., 2017).

RANKL is produced by VSMCs and endothelial cells, and transformation can promote the calcification of VSMCs and osteogenic differentiation. Leptin (Figure 3) partially promotes the differentiation of VSMCs into osteoblasts in female mice through the OB-Rb/PI3K/Akt/RANKL-bone morphogenetic protein-4 (BMP-4) pathway (Liu et al., 2014). The glucagon-like peptide-1 (GLP-1) analog Lilaru peptide (Figure 3) activates the GLP-1 receptor and inhibits the PI3K/Akt/mTOR/S6K1 signaling pathway, inhibiting osteogenic differentiation and the calcification of human VSMCs (Zhan et al., 2015).

Conversely, numerous studies have shown that activated PI3K/Akt can inhibit the osteogenic differentiation of VSMCs. Omentin-1 stimulated OPG expression in VSMCs and osteoblasts through the PI3K/Akt signaling pathway *in vitro*, inhibiting the production of RANKL, and alleviating arterial calcification in mice lacking OPG (Xie et al., 2011). Collett et al. showed that the Axl/PI3K/Akt pathway directly regulates the stromal mineralization of VSMCs through *in vitro* experiments and showed that targeting the receptor tyrosine kinase Axl receptor maintained high levels of Axl and key downstream PI3K/Akt signal transduction, inhibitings mineral deposition for the treatment of calcification (Collett et al., 2007). Inflammatory stimulation may also promote vascular calcification. Okazaki et al. showed that the PI3K/Akt pathway may play an inhibitory role in inflammatory mediators-induced calcium in human VSMCs by regulating alkaline phosphatase expression (Okazaki et al., 2009). Farnesyl transferase inhibitor-277 inhibits (FTI-277) phosphate-induced VSMC mineral deposition, prevents the osteogenic differentiation of VSMCs, and increases matrix Gla protein and mRNA expression by upregulating PI3K/Akt signaling and preventing cell apoptosis. This finding suggests that the targeted farnesylation or AKT may have therapeutic potential to prevent vascular calcification (Ponnusamy et al., 2018). Thus, PI3K plays a bidirectional regulatory role in calcification due to the different upstream signals of PI3K or differences in the experimental microenvironment.

### PI3K in Atherosclerotic Plaque Regression

In recent years, studies have shown that atherosclerotic plaque formation is reversible, and the main pathological changes associated with plaque regression include a reduction in foam cells, an increase in fibrous cap SMCs and the enhancement of plaque stability (Barrett, 2020). Foam cell reduction is the most important mechanism for atherosclerotic plaque regression and stabilization, and macrophage cholesterol transport is a critical player in foam cell formation (Vainio and Ikonen, 2003) According to recent research findings, the PI3K signaling pathway mainly affects lipid output in foam cells, reduces the formation of foam cells and promotes the migration of lipid and foam cells out of plaques.

Liver X receptor agonists promoted regression in LDLR^−/−^ mice, liver X receptor stimulated macrophage cholesterol efflux through the expression of ApoE and ABCA1, and the use of the PI3K inhibitor LY294002 could attenuate these effects. The lipid core is the most important component of atherosclerotic lesions. The size of the lipid core directly determines the stability and harmfulness of the plaque. The size of the lipid core ina plaque depends on the dynamic balance between lipids inside and outside the lesion of the artery (Sakakura et al., 2013). However, the reduction in plaque lipid core size caused by lowering cholesterol content alone is limited, and the enhancement of reverse cholesterol transport more effectively promotes plaque regression.

Many studies have shown that ABCA1 plays an important inhibitory role in the formation of foam cells and that regulating the expression of ABCA1 at least partially inhibits the progression of atherosclerosis. Sp1 is a transcription factor that controls the transcription of the ABCA1 gene. Phosphorylation of Sp1 enhances its binding to the ABCA1 promoter region and increases the expression of ABCA1 (Chistiakov et al., 2016). In THP-1 macrophages, through PI3K/PKCζ/Sp1, heat shock protein 27 increases the expression of ABCA1, thus promoting the outflow of macrophage cholesterol, reducing the accumulation of cholesterol in macrophages, and preventing the development of foam cells and macrophages to reduce the formation of atherosclerotic plaques (Kuang et al., 2017).

Impaired lymphatic drainage of the arterial wall can lead to intimal lipid accumulation and atherosclerosis. New research has found that cholesterol can be exported through lymphatic vessels. One study showed that R-Spondin 2 inhibits PI3K/AKT/eNOS signaling by R-Spondin 2 receptor LGR4 and inhibits lymphangiogenesis, which may provide evidence for new therapeutic strategies to promote lymphangiogenesis and improve cholesterol efflux from atherosclerosis (Singla et al., 2020).

In addition, selective inhibition of the PI3K/Akt/mTOR signaling pathway can induce autophagy in macrophages and is beneficial for regulating the outflow of cholesterol in macrophage foam cells. Results have shown that photodynamic therapy promoted cholesterol efflux by inducing autophagy, and autophagy partially promoted cholesterol efflux through the ROS/PI3K/Akt/mTOR signaling pathway in THP-1 and peritoneal macrophage-derived foam cells (Han et al., 2017). The migration of dendritic cells can also promote plaque regression. In cholesterol-loaded dendritic cells, adrenaline signaling through AR-β1 inhibits the expression of *ß*-Arrestin 2, resulting in decreases in PI3K phosphorylation, CCR7 expression and the secretion of MMP-9. Bisoprolol (an AR-β inhibitor) inhibited this process and promoted dendritic cell migration. However, since this process may lead to the proliferation and migration of SMCs, exacerbating atherosclerosis, bisoprolol may play dual roles in the development of atherosclerosis (Yang et al., 2013).

## PI3K-Targeted Therapy in Atherosclerosis

### PI3K and Inflammation

Atherosclerosis is a chronic inflammatory reaction in the vascular wall. Endothelial cells are damaged, releasing a large number of inflammatory factors and adhesion molecules and attracting inflammatory cells. The developing inflammatory response damages endothelial function, promotes the injury of SMCs, and ultimately accelerates the progression of atherosclerosis. Therefore, reducing the inflammatory response in atherosclerosis may be an effective strategy for the treatment of atherosclerosis (Hansson et al., 2006).

PI3Ks are important mediators of the signaling cascade associated with the inflammatory response, and PI3K controls two events mediated by selectin: capture and rolling. In addition, PI3K is essential for macrophage activation, including the stimulation of its upstream factor: ox-LDL, angiotensin II, and different chemokines (Campbell and Trimble, 2004). Deletion of the PI3K gene in the ApoE^−/−^ atherosclerosis model effectively reduced plaque size (Chang et al., 2007). Thus, blocking PI3K may be a promising and innovative treatment strategy.

PI3K/Akt signaling pathways enable the transcription factor NF-κB to enhance the activity of inflammatory mediator genes. Normally, NF-κB is present in the cytoplasm and is bound to the IκB family of inhibitory proteins, such as IκB-α and IκB-β, in unstimulated cells. However, activated IKKs phosphorylate IκBs, leading to IκB ubiquitination and proteasomal degradation. NF-κB is then released and transported to the nucleus, where it activates the transcription of target genes, including inflammatory cytokines and growth factors. Tumor necrosis factor-α (TNF-α) is an important cytokine associated with atherosclerosis progression.

Long-term exposure to high glucose or insulin can induce endothelial cells to express adhesion molecules. Insulin promotes the surface expression of Mac-1 through the PI3K/Akt signaling pathway and promotes monocyte adhesion to and subsequent migration across endothelial cells, which is a key process in atherosclerosis-associated chronic inflammation (Jin et al., 2014). TNF-α stimulates the release of inflammatory factors by regulating the PI3K/Akt and NF-κB signaling pathways, causing endothelial cell damage. However, total saponins inhibited these signaling pathways and were found to play a protective role in endothelial cells (Zhou et al., 2018). Defibrotide may play a protective role in the endothelium by inhibiting the upregulation of histone deacetylase through the PI3K/AKT signaling pathway and hindering endothelial cell transformation into a proinflammatory phenotypes, thus reducing the expression of adhesion receptors and decreasing oxidative stress (Shakespear et al., 2011; Palomo et al., 2020).

In atherosclerotic plaques, aortic CD4+T cells are mainly IFN- γ helper 1 cells, which secrete the pro-atherogenic cytokine IFN-γ to promote the occurrence of atherosclerosis, while Treg cells can inhibit the inflammatory response. Activation of mTOR signaling initiates the differentiation of effector factor CD4+T cells, increasing the percentage of Th1 cells and decreasing that of Treg cells. A recent study found that lymphocyte-specific protein tyrosine kinase inhibitors could reduce the Th1/Treg ratio by inhibiting PI3K/AKT/mTOR signaling, which reduced the inflammatory response, and enhanced plaque stability (Liu et al., 2020).

Apolipoproteins plays a role in regulating the development of internal inflammation in dermatitis. ApoM-S1P significantly downregulates the expression of proinflammatory cytokines, adhesion molecules and related proteins by activating downstream PI3K/AKT in combination with S1P receptor subtype 2 (S1PR2) to inhibit the ox-LDL-induced inflammatory response in human endothelial cells (Ruiz et al., 2017; Zheng et al., 2019), However, the another study showed that ApoA-1/SR-BI regulates S1P/S1PR2- induced human endothelial cells inflammation through the PI3K/Akt/eNOS/NF-κB signaling pathway (Kimura et al., 2006; Ren et al., 2017).

Retinoic acid inhibits tissue factor, plasminogen activator inhibitor-1 and high mobility group box-1 expression by regulating adenosine monophosphate-activated protein kinase (AMPK) activity in activated endothelial cells, and AMPK activity can be regulated by PI3K/Akt (Kim et al., 2015). The novel pentacyclic triterpenoid compound ilexgenin A can inhibit PI3K/Akt/NF-κB and MAPK-mediated inflammatory responses, which are characterized by cytokine production by THP-1 cells. Because of its oral safety profile, ilexgenin A has become a potential candidate for the clinical treatment of atherosclerosis (Liu et al., 2016). Saikosaponin-a induces an anti-immune inflammatory response by regulating the PI3K/AKT/NF-κB/nod-like receptor protein pathways. In addition, saikosaponin-a can inhibit the uptake of ox-LDL and the formation of foam cells by reducing the expression of LOX-1 and CD36 (He et al., 2016).

### PI3K and Endothelial Cells

Endothelial cells are a biological barrier between the blood and vascular walls. Endothelial cell dysfunction is the initiating factor for atherosclerosis (Cahill and Redmond, 2016), which is characterized by inflammatory processes, reduced NO bioavailability, and increased oxidative stress. After exposure to harmful factors, endothelial cells exhibit a series of changes, including increased permeability, reduced integrity, increased proinflammatory factors and reduced anti-inflammatory factors, which are collectively referred to as endothelial dysfunction and eventually leads to apoptosis (Gimbrone and García-Cardeña, 2016).

Since endothelial cell damage requires endothelial progenitor cell (EPC) proliferation, repair and replacement, many scholars have begun to explore the role of these cells in atherosclerosis progression. Increasing the number, regeneration and function of EPCs could promote the repair of damaged endothelial cell monolayers and inhibit mesangial SMC activity and neointima formation, thereby inhibiting atherosclerosis; statins and olmesartan mobilized EPCs in the bone marrow and inhibited endothelial progenitor cell apoptosis through the PI3K/Akt pathways, increasing the number of EPCs in the blood circulation and repairing damaged endothelial cells (Dimmeler et al., 2001; Gong et al., 2015). Reverse-D-4F improved high-fat diet-induced bone marrow-derived EPC functions in C57BL/6J mice via the PI3K/AKT/eNOS pathways, partially restoring TNF-α-induced EPC dysfunction (Nana et al., 2015).

#### Oxidative Stress

Vascular endothelial cell uptake of ox-LDL is a critical step in the development and progression of atherosclerosis. ox-LDL is the main factor leading to endothelial dysfunction and can induce autophagy and thus lead to atherosclerosis (Pirillo et al., 2013). When vascular endothelial cells encounter ox-LDL, PKB dephosphorylation inhibits the PI3K/Akt signaling pathway, resulting in increased apoptosis, decreased endothelial nitric oxide synthase activity, decreased cell migration, and damage to endothelial cells.

Ox-LDL-induced ROS-mediated oxidative stress in endothelial cells further damages endothelial cells. Studies have shown that ROS formation can lead to endothelial dysfunction by activating NADPH oxidase and eNOS uncoupling, and NADPH oxidase is the main enzyme in ROS production.7,8-Dihydroxy-3-methyl-isochromanone-4 (XJP-1) can protect (Figure 1)against ox-LDL-induced cytotoxicity and apoptosis by inhibiting ox-LDL-induced ROS production and increasing the production of NO through the PI3K/Akt/eNOS pathway (Fu et al., 2013). The antioxidant activity of Klotho can be mediated by activating the PI3K/Akt/eNOS pathway to upregulate oxidative scavenging agents and downregulate the expression of LOX-1, reversing the ox-LDL-induced inhibition of NO production (Yao et al., 2017) (Figure 1). Ginkgolide B reduces the expression of ox-LDL-stimulated endothelial cell junctional adhesion molecule-A, Cx43 and vascular endothelial cadherin through the PI3K/Akt pathway, reducing the permeability of the vascular wall, adhesion molecule expression and leukocyte migration (Liu et al., 2015; Hashimoto et al., 2007).

Endogenous antioxidant defense systems play a vital role in protecting vascular endothelial cells from oxidative stress damage. Activation of PI3K/Akt and up-regulation of heme oxygenase-1 (HO-1) might be new targets for inhibiting ox-LDL-induced apoptosis. Lunasin (Figure 1) is upregulated through the PI3K/Akt/Nrf2/antioxidant response element (ARE) pathway, and HO-1, attenuated oxidative-induced endothelial injury and inhibits the progression of atherosclerotic plaques in ApoE^−/−^ mice (Gu et al., 2019). Clinical trials have shown that the PI3K/Akt/Nrf2 signaling pathway plays an important role in cardiovascular cell antioxidants mediated by the metabolite S- (-) equol, which is derived from soybean isoflavones (Zhang et al., 2013). Lu et al. confirmed that andrographolide inhibits the formation of Src-driven NADPH complex (Figure 1),Upregulation of HO-1 and glutamate cysteine ligase modifier subunit gene expression, through the PI3K/Akt/Nrf2 and PI3K/Akt/AP-1 pathways increased glutathione levels, thus inhibiting TNF-α-induced ROS generation and intercellular adhesion molecule 1 (ICAM-1) expression (Lu et al., 2014).

#### Endothelial Cell Dysfunction

Endothelial cell dysfunction also plays a key role in the pathogenesis of atherosclerosis. Endothelial cell apoptosis impairs endothelial integrity and barrier function, ultimately leading the endothelial cells' to lose their ability to regulate lipid homeostasis, inflammation, and immunity (Gimbrone and García-Cardeña, 2016). Endothelial apoptosis can be reversed to protect and improve atherosclerosis. Recently, increasing evidence has indicated that the activation of PI3K/Akt/eNOS signaling coordinates protection against endothelial dysfunction and apoptosis. eNOS plays a key role in endothelial cell function and survival and is activated by PI3K/Akt-mediated phosphorylation to produce NO. An increase in luteinizing hormone can reduce the synthesis of NO by inhibiting this pathway and promoting the formation of atherosclerotic plaques (Meng et al., 2019). Overexpression of T-cadherin in endothelial cells attenuated insulin-induced PI3K/Akt/mTOR pathway activation and simultaneously reduced insulin-stimulated eNOS activation, migration, and angiogenesis, suggesting the role of endothelial dysfunction induced by T-cadherin in atherosclerosis (Philippova et al., 2012).

Recently, C1q/tumor necrosis factor-related protein-3 (CTRP3) was found to mediates the inhibition of ox-LDL-induced PI3K/Akt/eNOS phosphorylation (Chen et al., 2019). Clinical trials have shown that activation of the AMPK/PI3K/Akt/eNOS pathway through GLP-1R/cAMP significantly improved endothelial function in coronary arteries in newly diagnosed type 2 diabetes patients (Wei et al., 2016). Thymic stromal lymphopoietin activates HOTAIR (a lncRNA) transcription through the PI3K/AKT/IRF1 pathways, thus regulating endothelial cell proliferation and migration (Peng et al., 2017).

Many factors promote vascular endothelial cell apoptosis, such as cytokines, ROS, lipopolysaccharide and inflammation, in which oxidative stress induces increased mitochondrial membrane permeability and cytoplasmic release of cytochrome, triggering apoptosis through caspase 9/3 signaling cascades. The latest results suggest that gypenosides may regulate mitochondrial-mediated apoptosis in ApoE^−/−^ mice through the PI3K/Akt/Bad pathways, thus inhibiting atherosclerosis (Song et al., 2020). In addition, the PI3K/AKT/FOXO3A signaling pathways plays important roles in apoptosis; FOXO3a is an important downstream effector of the PI3K/AKT pathway, and activated AKT can inhibit FOXO3a nuclear transcription and apoptosis. Scutellarin (Figure 1) may inhibit vascular endothelial cell injury and apoptosis by regulating the PI3K/AKT/FOXO3a signaling pathways (Fu et al., 2019).

In recent years, new discoveries on the influence of PI3K on atherosclerosis in terms of physical factors have been made. Laminar flow inhibits the unfolded protein response and endoplasmic reticulum stress-induced endothelial cell apoptosis through the PI3K/Akt signaling pathway, providing a protective effect against atherosclerosis (Kim and Woo, 2018). Autophagy also plays a protective role in endothelial cells in the context of atherosclerosis, reducing endothelial cell apoptosis. Kaempferol (Figure 1) enhances autophagy by inhibiting the PI3K/Akt/mTOR pathways in human endothelial cells, thereby inhibiting ox-LDL-induced apoptosis (Che et al., 2017). Gossyphenol could mediate autophagy through the Class III PI3K/beclin-1 and PTEN/Class I PI3K/Akt signaling cascades to improve atherosclerotic lesions and ox-LDL-induced endothelial injury *in vivo* (Lin et al., 2015).

### PI3K and SMCs

The proliferation of VSMCs plays a crucial role in atherosclerosis. SMCs are the major cell types in the vascular wall, and their primary role is to maintain vascular tension and vascular homeostasis. In a healthy state, SMCs are in a state of contraction, differentiation and low turnover (Frismantiene et al., 2018), but after injury, these cells transform into a synthetic phenotype stimulated by circulating cytokines and growth factors and characterized by increased proliferation, the loss of contractility and the secretion of ECM degrading enzymes. This phenotypic change allows SMCs to migrate to the intima of blood vessels, triggering an adaptive response that ultimately exacerbates the development of intimal hyperplasia and restenosis (Bennett et al., 2016). By regulating the abnormal proliferation and migration of VSMCs, PI3K leads to thickening of the arterial intima, which is an important step in the development of atherosclerosis.

Platelet-derived growth factor BB (PDGF-BB) is an effective SMC mitogen and chemokine that can induce the dedifferentiation of cells into a synthetic phenotype and increase proliferation and migration to the intima. In addition to being secreted by platelets, PDGF is also secreted by endothelial cells and SMCs. PDGF-BB induced differentiation of SMCs is driven by the PI3K/Akt and MAPK/ERK1/2 intracellular transduction pathways (Kim and Yun, 2007). Ashwani et al. found that PDGF-BB could induce the expression of miR-21 in human saphenous veins- derived SMCs through the PI3K and ERK signaling pathways, subsequently driving the SMC phenotype, thereby promoting intimal proliferation (Alshanwani et al., 2018). CTRP6 (Figure 3) inhibits PDGF-BB-induced proliferation and migration of VSMCs by inhibiting the PI3K/Akt/mTOR signaling pathway (Dong et al., 2018). Wang et al. showed that exchange proteins directly activated by cAMP isoform 1 promoted neointimal formation by promoting PI3K/AKT signal transduction in VSMCs and verified that Epac1 inhibitors can be used as effective treatments for vascular proliferative diseases (Wang et al., 2016).

Migration is a complex vascular response that includes chemotaxis, motility and invasion. Studies have shown that MMP-9 expression and cellular migration are mediated by activation of the PI3K/Akt signaling pathways, playing key roles in ECM degradation and VSMC invasion and migration. Mulberry pigment can effectively inhibit the activity of PDGF-induced MMP-9 by downregulating the binding activity of NF-κB, thus reducing the migration and invasion of SMCs (Shin et al., 2018). Naringin (Figure 3) at concentrations of 10–25 μM blocked the TNF-α-induced PI3K/AKT/mTOR/p70S6K pathway in VSMCs at concentrations of 10–25 μM, and the expression of MMP-9 was inhibited by the transcription factors NF-κB and AP-1 (Lee et al., 2009).

The synergistic effects of atorvastatin and cyanidin-3-glucoside improves HASMC proliferation and migration by enhancing cell cycle arrest through the AT 1R-mediated MAPK and PI3K/Akt pathways (Pantan et al., 2016). FOXO3a phosphorylation plays an important role in mediating MMP-2 activation and VSMC migration. In the osteogenic differentiation of SMCs, apelin (Figure 3) inhibits osteogenic differentiation by activating PI3K/Akt (Shan et al., 2011). However, activating PI3K/Akt/FoxO3a/MMP-2 and promoting the degradation of the basement membrane and ECM led to VSMC migration (Wang C. et al., 2015).

The interaction of advanced glycation end products (AGEs) and receptor of advanced glycation end product (RAGE) (Figure 3) in the vascular system of diabetic patients activates downstream PI3K/AKT pathways, thereby promoting human aorta VSMC proliferation and migration. Consequently, inhibition of the PI3K/AKT pathway may become a new strategy for the treatment of diabetic atherosclerosis (Yuan et al., 2020). Rosiglitazone (Figure 3) has been reported to inhibit insulin-stimulated VSMC proliferation by inhibiting the PI3K/Akt/mTOR/P70S6K cascades (Park et al., 2008). Activation of VSMC NF-κB in hyperglycemia is a key mechanism by which VSMC factor production mediates diabetic vasculr complications. Rutin significantly reduced the proliferation and migration of VSMCs in obese type 2 diabetic rats induced by intermittent hyperglycemia by reducing PI3K and NF-κB phosphorylation (Yu et al., 2016).

Some microRNA (miRNA) can affect the development of atherosclerosis through PI3K signaling pathway, so miRNA may be candidate drugs for PI3K target drugs. MiR-145 can inhibit VSMC proliferation, migration and phenotypic transformation by blocking the activation of PI3K/Akt/mTOR signaling pathway (Zhang et al., 2020). However, miR-647 promotes the proliferation and migration of HA-VSMC treated with OX-LDL at least in part by targeting the PTEN/PI3K/AKT pathway. Therefore, lowering miR-647 may be an effective method for the treatment of atherosclerotic tube wall thickening (Xu C. et al., 2019).

Recent studies have shown that IGF-1 is involved in protein synthesis and cell migration and proliferation and plays an important role in inhibiting the apoptosis of vascular smooth muscle cells, fibroblasts and macrophages. Tanshinone I (Figure 3) inhibits vascular smooth muscle cell proliferation by targeting the IGF-1 receptor/PI3K signaling pathway (Jia et al., 2010; Wu Y. T. et al., 2019). Dopamine D1-like receptor (Figure 3) activation inhibits IGF-1 in the context of vascular smooth muscle cell proliferation by inhibiting the IGF-1R/Akt/mTOR/p70S6K pathways and downregulating IGF-1 receptor expression (Kou et al., 2014).

VSMCs are also an important source of foam cells in atherosclerosis. Promoting the outflow of intracellular cholesterol and thereby reducing the formation of foam cells derived from VSMCs has become a treatment direction for atherosclerosis (Pryma et al., 2019). A recent study found that activation of P2RY12 receptor activated mTOR through PI3K/AKT to block autophagy in advanced atherosclerosis and reduce cholesterol outflow (Pi et al., 2020). However, overexpression of miR-223 could induce autophagy and inhibit the formation of foam cells in VSMCs by reducing IGF-1R/PI3K/Akt signal transduction (Wu W. et al., 2019) PI3K/AKT mediated autophagy in VSMCs may be a targeted pathway to reduce foam cell formation.

### PI3K and Macrophages

In the atherosclerotic lesions of mice with ox-LDL, atherosclerotic chemokines, angiotensin II, and hypercholesterolemia, PI3K P110γ deletion reduced macrophage proliferation by inhibiting activation of the PI3K/Akt pathway in macrophages (Zotes et al., 2013). AngiogeninI, which is located upstream of PI3K, induces monocyte chemotaxis, and MCP-1 is a potent monocyte chemokine. By promoting the infiltration of monocytes into the intima, MCP-1 plays a key role in early atherosclerosis development.

Elevated expression of the chemokine CXCL8 is mediated by peptidoglycan and triggers monocyte arrest in early atherosclerotic endothelial cells, which can be significantly attenuated by inhibitors of the PI3K/Akt/mTOR pathways (Lee et al., 2015). Isorhamnetin reduces apoptosis by significantly inhibiting ox-LDL-induced caspase-3 activation, activating PI3K/AKT/Nrf2, increasing the expression of HO-1, reducing the level of ROS and lipid deposition of macrophages, reducing the size of atherosclerotic plaques, and reducing the accumulation of apoptotic macrophages in lesions (Luo et al., 2015). Heat-killed *S. aureus* therapy can prevent the development of atherosclerosis by inducing a strong anti-inflammatory IL-10 response by macrophages (Frodermann et al., 2016). PI3K may be involved in the determination of macrophage phenotypes, and increased PI3K signaling has been found to bias macrophages toward the M2 phenotype (Linton et al., 2016).

ACAT-1 plays a key role in the formation of foam cells by catalyzing the esterification of free cholesterol to form cytoplasmic lipid droplets. Matsuo K et al. identified a new protein and showed that ARIA (Figure 2) deficiency attenuated the activity of PTEN, thereby enhancing the activation of PI3K/Akt, reducing the expression of ACAT-1 and reducing the formation of foam cells (Matsuo et al., 2015). Wang J demonstrated that *in vitro*, miR-223 (Figure 2) reversed macrophage-mediated lipid deposition and the inflammatory response by activating PI3K/AKT-mediated TLR4-NF-κB signaling (Wang J. et al., 2015). LRP1 regulates LXR-driven ABCA1 gene expression through the SHC1/PI3K/Akt/PPARγ/LXRα axis. Due to the anti-inflammatory properties of ABCA1, it can effectively maintain the cholesterol homeostasis in mouse macrophages and inhibit the inflammatory response of atherosclerosis (Tang et al., 2009; Xian et al., 2017).

Growing evidence has shown that PKC subtypes may be involved in atherosclerosis (Figure 2). PKCδ and its associated downstream signaling molecules, including activated EKR and activated Akt, were demonstrated to be highly expressed in human atherosclerotic plaques and infiltrated CD68-positive macrophages for the first time. PKCδ knockdown attenuated the activity of PI3K/Akt, reducing the expression of SR-A and CD-36. Therefore, down-regulation of PKCδ might be beneficial for the treatment of atherosclerosis (Lin et al., 2012).

Cholesterol efflux transfers cholesterol from macrophages to high-density lipoprotein particles and promotes the removal of excess cholesterol from the blood to the liver for excretion into bile. Cholesterol efflux capacity has been considered to be a predictor of atherosclerotic burden (Chistiakov et al., 2016). Xu X et al. provided evidence that macrophage cholesterol efflux is directly associated with endoplasmic reticulum stress and found that naringenin (Figure 2) promoted macrophage cholesterol efflux by activating PI3K/AKT and inhibiting ER stress-induced ATF6 activity (Xu X. et al., 2019). Tetramethyl pyrazine downregulates scavenger receptors and upregulates ATP-binding box transporters through PI3K/Akt and p38/Mapk signaling, thereby inhibiting lipid accumulation in macrophages (Duan et al., 2017). Oligomeric proanthocyanidins (OPCs) and epigallocatechin gallate (EGCG) promote cholesterol efflux in macrophage foam cells by activating the III PI3K/Beclin-1-mediated autophagy pathway, which may alleviate the pathological process of atherosclerosis (Jamuna et al., 2019).

Macrophages have of thrombospondin motif genes, which encode proteases involved in ECM remodeling and play an important role in the vulnerability of atherosclerotic plaques. Lauric acid was found to interfere with the expression of thrombospondin motifs through the PI3K/JNK pathway to prevent thrombosis (Ong et al., 2019). Another mechanism of macrophage foam cell formation is fluid-phase pinocytosis of LDL by macrophages. Recent studies have shown that PI3K mediates the macrophage macropinocytosis of LDL; therefore, PI3K may be a relevant target for inhibiting macrophage cholesterol accumulation in atherosclerosis (Kruth, 2013).

### PI3K and Platelets

Platelet activation and subsequent aggregation are complex and critical processes involved in thrombus formation after atherosclerotic plaque ruptures. Searching for drugs that can block platelet aggregation is important for treating atherosclerosis (Durrant and Hers,2020b).

Although the signaling pathways leading to the secretion of platelet particles are unclear, some studies have shown that PI3K is the central molecule associated with intracellular signaling in platelets. For a long time, the prevailing view was that NO inhibits platelet activation by increasing cyclic guanosine monophosphate levels. Recent studies have shown that PI3K/Akt signaling mediates platelet granule secretion mainly by activating the eNOS/NO cyclic GMP pathway and subsequently activating MAPK. Anthocyanins can effectively inhibit the secretion of platelet particles by inhibiting the PI3K/Akt/eNOS/NO/cGMP signaling pathways, thereby inhibiting MAPK activation in platelets. Therefore, oral anthocyanins may be an effective therapeutic strategy to prevent thrombosis and atherosclerosis in patients with hypercholesterolemia (Song et al., 2014). Statins induce tyrosine phosphorylation by activating platelet endothelial cell adhesion molecule-1 signaling. Recruitment and activation of the tyrosine phosphatase SHP-2 lead to its binding to PI3K and attenuates PI3K signaling, inhibiting Akt activation and thus platelet activation (Moraes et al., 2013).

The above studies have shown that inhibiting PI3K/Akt activity can inhibit platelet activation, but some studies have found that activating PI3K and inhibiting autophagy can also reduce platelet activation. Ox-LDL-induced oxidative stress triggers platelet activation and increases ROS, thereby inhibiting the activity downstream of the PI3K/Akt/mTOR signaling pathway, promoting platelet autophagy and exacerbating platelet aggregation. Wang et al. demonstrated experimentally that ROS inhibitors could improve this phenomenon by activating this signal and inhibiting autophagy (Wang et al., 2018).

### PI3K in Myocardial Infarction

The rupture of plaques in coronary arteries leads to the formation of occlusive thrombosis, which blocks blood flow in the artery and leads to acute cardiac ischemia and myocardial infarction (MI) (Palasubramaniam et al., 2019), one of the most important complications of atherosclerosis. PI3K not only can prevent MI by preventing plaque rupture and platelet aggregation, but also plays a unique role in the follow-up treatment of MI, especially in the area of ischemia reperfusion injury.

Activation of PI3K (p110α) protects the heart from MI induced heart failure (Lin et al., 2010). PI3K/Akt signaling can reduce apoptosis induced by myocardial ischemia/reperfusion. Nanoparticle-mediated pitavastatin protects the myocardium by targeting this signaling pathway (Nagaoka et al., 2015). Mesenchymal stem cell-derived exocrine activates the PI3K/Akt pathway, enhances myocardial activity and preventings adverse remodeling (Arslan et al., 2013). Eplerenone significantly reduced MI in diabetic rats through PI3K/Akt/GSK-3β (Mahajan et al., 2018). In addition, different PI3K signaling pathways have become ideal targets for inhibiting MI. For example, geraniol and levosimendan regulate autophagy to reduce oxidative stress and limit the inflammation cascade through PI3K/Akt/mTOR. Because levosimendan can inhibit aggregation, it is more suitable for hypercoagulation and hyperlipidemia (Tawfik et al., 2018; Younis et al., 2020).

Baicalin promotes NO production in cardiac microvascular endothelial cells through PI3K/Akt/eNOS and reduces ischemia/reperfusion injury (Bai et al., 2019). In addition, reconstituted HDL (rHDL) activates PI3K/Akt in monocytes/EPCs, thereby promoting their differentiation into endothelial-like cells. eNOS plays a crucial role in the angiogenesis of rHDL, which in turn can promote ischemic disease-induced angiogenesis through the activation of circulating EPCs and eNOS in preexisting endothelial cells (Sumi et al., 2007). Notably, although PI3Kγ inhibitors act as anti-inflammatory and anti-atherosclerosis agents, and the use of PI3Kγ inhibitors can affect the prognosis of MI due to the critical role of these inhibitors in the repair of angiogenesis in MI (Siragusa et al., 2010).

## Conclusion and Perspectives

In summary, PI3K and its signaling pathways are important regulators of atherosclerosis. Under different pathological conditions, PI3K is activated by the corresponding upstream pathway, subsequently activating downstream signal transduction. On the one hand, PI3K regulates the expression of related target genes and the proliferation, differentiation, apoptosis and migration of cells. On the other hand, PI3K further regulates other signaling pathways in a positive or negative way, indirectly affecting the development of atherosclerosis.

PI3K plays an active role in the survival and function of endothelial cells and EPCs, activates the PI3K/Akt/eNOS and PI3K/Akt/Nrf2/ARE pathways, and may be an ideal target to protect endothelial cells against apoptosis and oxidation. In SMCs, PI3K/Akt/mTOR is a classic downstream pathways of PDGF-BB. Excessive activation can lead to cell proliferation and thickening, leading to lumen stenosis and increasing the risks of vascular stenosis and obstruction. In macrophages, the progression and regression of plaques are affected by the regulation of lipid balance within and outside the cell, and macrophage apoptosis affects inflammation. In the context of plaque calcification, the PI3K signaling pathway is subject to different upstream activation signals and plays positive and negative regulatory roles.

PI3K and its signaling pathways are often essential links in the inflammatory response. On the one hand, PI3K signaling promotes the adhesion of endothelial cells and monocytes, and on the other hand, PI3K signaling can promote the expression of inflammatory molecules and mediators. Recent studies have found that PI3K reduces inflammation by negatively regulating TLR4 signaling, indicating a new role for PI3K in inflammation. The study of PI3K-related signaling pathways provides new ideas for clinical anti-inflammatory treatments by reducing oxidative stress and promoting intracellular cholesterol lipid transport out of cells.

Activators or inhibitors of PI3K and its signaling pathways are at an advanced stage of development in basic research, and drugs targeting PI3K and its related signaling pathways are promising treatments for atherosclerosis (Table 1), but currently, there is a lack of sufficient *in vivo* analysis and clinical trials to test the clinical efficacy of these drugs are lacking. Therefore, there is still a long way to go before PI3K can be applied in the clinic to bring real benefits to patients with atherosclerosis. A large number of results have been obtained in preclinical studies of PI3K-related regulators in cardiovascular diseases and tumors. For example, phase I clinical trials have shown that CUDC-907, a dual inhibitor of histone deacetylase and PI3K, has good activity in the treatment of recurrent or refractory lymphoma and multiple myeloma (Younes et al., 2016). Furthermore, amlodipine besylate combined with acupoint Chinese medicine can reduce the expression of VEGF and MMP-9 through the PI3K/Akt pathway and improve renal failure and hypertension (Feng et al., 2019).

**TABLE 1:** Targeted drugs of PI3K and its signaling pathway in atherosclerosis.

References	Signaling pathways	Target drugs	Effect
Endothelial cell			
Zhou et al. (2018)	PI3K/Akt/NF-κB	Total saponins	Inhibit inflammation
Palomo et al. (2020)	PI3K/Akt	Defibrotide	Hinder endothelial cell transformation into proinflammatory phenotypes
Kim et al. (2015)	PI3K/Akt	Retinoic acid	Inhibit inflammation by regulating AMPK
Liu et al. (2015)	PI3K/Akt	Ginkgolide B	Reduce the permeability of the vascular wall
Fu et al. (2013)	PI3K/AKT/eNOS	XJP-1	Protect against ox-LDL-induced cytotoxicity and apoptosis
Chen et al. (2019)	PI3K/AKT/eNOS	CTRP3	Mediate the inhibition of ox-LDL
Nana et al. (2015)	PI3K/AKT/eNOS	Reverse-D-4F	Restore TNF-α-induced endothelial progenitor cell dysfunction
Lu et al. (2014)	PI3K/Akt/Nrf2 and PI3K/Akt/AP-1	Andrographolide	Increase glutathione levels and inhibit ICAM-1 expression
Gu et al. (2019)	PI3K/Akt/Nrf2	Lunasin	Attenuate oxidative-induced endothelial injury
Peng et al. (2017)	PI3K/AKT/IRF1	Thymic stromal lymphopoietin	Regulate EC proliferation and migration
Song et al. (2020)	PI3K/Akt/Bad	Gypenosides	Regulate mitochondrial-mediated apoptosis
Fu et al. (2019)	PI3K/AKT/FOXO3A	Scutellarin	Inhibit vascular endothelial cell injury and apoptosis
Lin et al. (2015)	III PI3K/Beclin-1 and PTEN/PI3K/Akt	Gossyphenol	Improve atherosclerotic lesions and ox-LDL-induced endothelial injury
Che et al. (2017)	PI3K/Akt/mTOR	Kaempferol	Enhance autophagy and inhibit ox-LDL-induced apoptosis
Smooth muscle cell			
Tang et al. (2015)	PI3K/AKT/Runx2	VPO1 inhibitor	Hinder ox-LDL-induced vascular calcification
Zhan et al. (2015)	PI3K/Akt/mTOR/S6K1	Lilaru peptide	Inhibit osteogenic differentiation and the calcification
Xie et al. (2011)	PI3K/AKT	Omentin-1	Stimulate OPG expression and inhibit the production of RANKL
Ponnusamyet al. (2018)	PI3K/AKT	FTI-277	Prevent vascular calcification
Dong et al. (2018)	PI3K/Akt/mTOR	CTRP6	Inhibit PDGF-BB-induced proliferation and migration
Wang et al. (2016)	PI3K/AKT	Epac1 inhibitor	Inhibit neointimal formation
Pantan et al. (2016)	PI3K/AKT	atorvastatin and cyanidin-3-glucoside	Improves HASMC proliferation and migration
Shin et al. (2018)	PI3K/Akt/NF-κB	Mulberry pigment	Inhibit the activity of PDGF-induced MMP-9
Lee et al. (2009)	PI3K/AKT/mTOR/p70S6K	Naringin	Inhibit the expression of TNF-α-induced MMP-9
Park et al. (2008)	PI3K/AKT/mTOR/p70S6K	Rosiglitazone	Inhibit insulin-stimulated proliferation
Wu W. et al. (2019)	IGF-1R/PI3K/Akt	Overexpression of miR-223	Induce autophagy and inhibit the formation of foam cells
Wu Y. T. et al. (2019)	IGF-1R/PI3K	Tanshinone I	Inhibit vascular smooth muscle cell proliferation
Kou et al. (2014)	IGF-1R/Akt/mTOR/p70S6K	Dopamine D1-like receptor	Inhibit vascular smooth muscle cell proliferation
Macrophage			
Wu et al. (2020)	PI3K/Akt/mTOR	miR-135b	Enhance macrophage autophagy
Han et al. (2019)	PI3K/Akt/mTOR	PDT	Reduce inflammation and increase plaque stability
Han et al. (2017)	PI3K/Akt/mTOR	Photodynamic therapy	Promote cholesterol efflux by inducing autophagy
Zheng et al. (2020)	PI3K/AKT/TLR4	GYY4137	Improve foam cell formation and reduce the expression of proinflammatorycytokines
Wang et al. (2020)	PI3K/AKT/TLR4/NF-κB	Tanshinone IIA and Astragaloside IV	Inhibit the expression of MMP-9, stabilize plaque and anti-inflammatory
Endale et al. (2013)	PI3K/AKT/TLR4/NF-κB	Quercetin	Attenuate the inflammatory response
Wang J. et al. (2015)	PI3K/AKT/TLR4/NF-κB	miR-223	Reverse macrophage-mediated lipid deposition and the inflammatory response
Luo et al. (2015)	PI3K/AKT/Nrf2	Isorhamnetin	Increase the expression of HO-1 and reduce the level of ROS
Kuang et al. (2017)	PI3K/PKCζ/Sp1	HSP 27	Promote the outflow of macrophage cholesterol
Xu X. et al. (2019)	PI3K/Akt	Naringenin	Promote macrophage cholesterol efflux
Fougerat et al. (2008)	PI3K/Akt	PI3K P110γ deletion	Attenuate the inflammatory response
Jamuna et al. (2019)	III PI3K/Beclin-1	OPC and EGCG	Promote cholesterol efflux in macrophage foam cells
Matsuo et al. (2015)	PTEN/PI3K/Akt	ARIA	Reduce the expression of ACAT-1 and the formation of foam cells
Ong et al., 2019)	PI3K/JNK	Lauric acid	Interfere with the expression of thrombospondin motifs
Immune cells and immune inflammation			
Liu et al. (2020)	PI3K/Akt/mTOR	Lymphocyte-specific protein tyrosine kinase inhibitors	Reduce Th1/Treg ratio and inflammatory response
Lin et al. (2017)	Dab2/Src/PI3K/Akt/NF-κB	Quercetin	Attenuate DC activation
He et al. (2016)	PI3K/AKT/NF-κB/NLRP	Saikosaponin-a	Anti-immune inflammatory response
Platelet			
Song et al. (2014)	PI3K/Akt/eNOS/NO/cGMP	Anthocyanins	Inhibit MAPK activation in platelets
Moraes et al. (2013)	PI3K/Akt	Statins	Inhibit platelet activation
Wang et al. (2018)	PI3K/Akt/mTOR	ROS inhibitors	Inhibit platelet autophagy and alleviate platelet aggregation
Myocardial infarction			
Nagaoka et al. (2015)	PI3K/Akt	Nanoparticle-mediated pitavastatin	Protect the myocardium
Arslan et al. (2013)	PI3K/Akt	Mesenchymal stem cell-derived exocrine	Enhance myocardial activity
Mahajan et al. (2018)	PI3K/Akt/GSK-3β	Eplerenone	Reduce myocardial infarction
Younis et al. (2020)	PI3K/Akt/mTOR	Geraniol	Regulate autophagy to reduce oxidative stress
Bai et al. (2019)	PI3K/Akt/eNOS	Baicalin	Reduce ischemia/reperfusion injury
Sumi et al. (2007)	PI3K/Akt/eNOS	rHDL	Promote ischemic disease-induced angiogenesis

These findings provide support for further clinical study of PI3K in atherosclerosis. It is believed that with increasingly in-depth research on PI3K and the continuous exploration of PI3K and its signaling pathways, more specific mechanisms in atherosclerosis can be identified, and effective targeted drugs can be developed, bringing new strategies for the prevention and treatment of atherosclerosis to the clinic.

## Glossary

ABCA1: ATP-binding cassette transporter A1
AGEs: advanced glycation end products
AMPK: adenosine monophosphate-activated protein kinase
ARE: antioxidant response element
ATP: adenosine triphosphate
BMP: bone morphogenetic protein
CML: Nε-carboxymethyl-lysine
CTRP: C1q/tumor necrosis factor-related protein
eNOS: endothelial nitric oxide synthase
EPC: endothelial progenitor cell
ERK: extracellular regulated kinase
GDF: growth differentiation factor
GLP: glucagon-like peptide
HO-1: heme oxygenase-1
GSK3β: glycogen synthase kinase-3β
ICAM-1: intercellular adhesion molecule 1
IGF: insulin-like growth factor
LDL: Low-density lipoprotein
LOX-1: Lectin like Ox-LDL receptor-1
M1: the classical activated macrophage
MAPK: mitogen-activated protein kinase
MCP-1: monocyte chemoattractant protein-1
MMP: matrix metalloproteinases
mTOR: mammalian target of rapamycin
NADPH: nicotinamide adenine dinucleotide phosphate
NF-κB: nuclear factor-kappa B
Nrf2: nuclear factor erythroid 2-related factor 2
OPG: osteoprotegerin
OPN: osteopontin
ox-LDL: oxidized low-density lipoprotein
PI3K: Phosphatidylinositol 3 kinase
PAPPA: pregnancy-associated plasma protein A
PDK1: phosphoinositide-dependent kinase 1
PDGF-BB: Platelet-derived growth factor BB
PKB/Akt: protein kinase B
PKC: Protein kinase C
RAGE: receptor of advanced glycation end product
RANKL: receptor activator for nuclear factor-kB ligand
ROS: reactive oxygen species
Runx2: runt-related transcription factor 2
S6K1: S6 kinase 1
S1P: Sphingosine-1 phosphate
S1PR2: S1P receptor subtype 2
SMC: smooth muscle cell
SR-BI: Scavenger Receptor class B type I
TNF-α: Tumor necrosis factor-alpha
TLR4: Toll-like receptor 4
VEGF: vascular endothelial growth factor
XJP-1: 7,8-Dihydroxy-3-methyl-isochromanone-4b
